# Novel sporozoite-based ELISpot assay to assess frequency of parasite-specific B cells after vaccination with irradiated sporozoites

**DOI:** 10.1186/s12936-019-2819-6

**Published:** 2019-05-29

**Authors:** Tanmaya Atre, Tanisha M. Robinson, Tatyana Savransky, Sheetij Dutta, Judith E. Epstein, Elke S. Bergmann-Leitner

**Affiliations:** 1 Malaria Vaccine Branch, US Military Malaria Research Program, Walter Reed Army Institute of Research, Silver Spring, MD USA; 2 Division of Entomology, Walter Reed Army Institute of Research, Silver Spring, MD USA; 3 Malaria Department, Naval Medical Research Center, Silver Spring, MD USA

**Keywords:** Malaria, Sporozoite, Whole parasite vaccine, ELISpot, B cells, Antibodies, Immunity

## Abstract

**Background:**

Whole parasite vaccination is an efficacious strategy to induce sterile immunity and to prevent malaria transmission. Understanding the mechanism and response of immune cells to vaccines plays a critical role in deciphering correlates of protection against infection and disease. Immunoassays, such as ELISpot, are commonly used to assess the immunogenicity of vaccines towards T cells and B cells. To date, these assays only analyse responses to specific antigens since they are based on recombinant parasite-derived proteins or peptides. There is the need for an agnostic approach that allows the evaluation of all sporozoite-associated antigens.

**Methods:**

ELISpot plates coated with a defined amount of lysed *Plasmodium falciparum* sporozoites were used to assess the frequency of sporozoite-specific B cells in peripheral blood mononuclear cells from donors immunized with either a recombinant malaria vaccine or irradiated sporozoites.

**Results:**

This report describes the assay conditions for a specific and sensitive sporozoite-based B cell ELISpot assay. The assay development considers the quality of sporozoite preparation as well as the detection threshold of the frequency of antigen-specific B cells. The assay enables the detection of sporozoite-specific IgM and IgG-producing B cells. Moreover, the assay can detect sporozoite-reactive B cells from subjects that were either vaccinated with the radiation attenuated sporozoite vaccine or a recombinant pre-erythrocytic vaccine.

**Conclusion:**

The newly developed sporozoite-based B cell ELISpot enables the monitoring of changes in the frequency of sporozoite-specific B cells. Applying this assay to assess the potency of vaccination regimens or seasonal changes in B cell populations from subjects residing in malaria-endemic areas will provide an opportunity to gain insight into immune mechanisms involved in protection and/or disease.

## Background

Malaria parasites start their mammalian life cycle upon injection via mosquito bite into the host (reviewed in [1, 2]). Sporozoites migrate in the skin and most of them eventually either enter the lymphatic system or the blood stream by crossing the vascular endothelium. The sporozoites will arrive in the liver, the target site for sporozoite infection. Within the liver, a high replication of parasites occurs, where one infected hepatocyte will ultimately release up to 30,000 merozoites. This starts the erythrocytic stage of the infection, which is responsible for morbidity and, in case of *Plasmodium falciparum*, often mortality. Some of the erythrocytic parasites develop into sexual forms (gametocytes), which are ultimately picked up by mosquitoes during a blood meal thus completing the transmission cycle.

Recently, proteomic profiling revealed that *P. falciparum* sporozoites express 1991 proteins [3]. Current vaccine models include targeting surface proteins of the sporozoite laying the groundwork for sub-unit vaccines. Circumsporozoite protein (CSP) is the most studied vaccine target since it is the most abundantly expressed surface antigen on the sporozoite. The most promising sub-unit vaccine is the RTS,S/AS01 vaccine, which is a fusion of the hepatitis surface antigen (HBsAg) as carrier with the central repeat region and the C-terminus of CSP. This vaccine has achieved 50–86% protection against experimental sporozoite challenge depending on the vaccination regimen [4, 5]. Whole parasite vaccines (WPV) are attractive as they have the potential to induce immune responses against a wide range of antigens associated with the sporozoite. Attenuation is achieved through radiation attenuated (RAS) or genetically engineered sporozoites (GAS) or immunizing with non-attenuated parasites while treating vaccinees with anti-malarial drugs (reviewed in [6–8]). The oldest approach, the RAS vaccine, initially employed delivery through bites by irradiated mosquitoes and exposing study participants to a large number of bites in monthly intervals [9]. Attenuated sporozoites are able to infect the liver, but depending on the attenuation protocol, the parasites stall their development into infectious blood-stage parasites. Immunization with RAS leads to sterile protection that breaks the transmission cycle and is, therefore, considered the gold standard for developing recombinant or sub-unit vaccines.

The RAS vaccine has been reported to mediate protection through an array of immune mechanisms; antibodies, CD4^+^, CD8^+^ and γδ TCR T cells have all been shown to play a crucial role in protection [10, 11]. While there is a strong focus on the identification of cellular mechanisms, the characterization of sporozoite-specific B cells has been unable to garner similar enthusiasm even though preclinical models have identified a crucial role for B cells in parasite clearance [12, 13]. In humans, one study describes the induction of B cells after GAS vaccination [14]. Here, the B cells were tested in ELISpot assays with nine plasmodial antigens (of which four are expressed on the sporozoite). No comparable analysis has been reported so far with RAS-induced B cells.

B cell ELISpots are a relatively simple and robust method to measure the frequency of antibody-secreting memory B cells in peripheral blood mononuclear cells. ELISpot assays enable the quantification of antigen-specific B cells and can be considered complimentary to ELISA assays. While serum ELISA assays determine the titres of antigen-specific antibodies, they do not provide information about the number of antigen-specific B cells. Combining ELISA results with ELISpot data provides deeper insights into humoral immunity to vaccines. Recently, the development of a sporozoite-based ELISA method was reported [11]. It allows the detection of sporozoite-specific antibodies that a classic ELISA based on recombinant proteins or peptides failed to report. Similarly, the present study overcomes the need for recombinant proteins or peptides when measuring antigen-specific B cells that are specific for this particular antigen. In contrast, the method offers the opportunity to agnostically evaluate and quantify all sporozoite-specific B cells by using either intact or lysed sporozoites as plate antigens. Subsequent integration of ELISpot and ELISA data from these sporozoite-based assays will support an in-depth analysis of the frequency of vaccine induced, parasite specific B cell responses and the type of antibodies they secrete. The sensitivity of the assay makes it possible to perform the analysis even when only a limited cell number is available.

## Methods

### Parasite strain and reagents

*Plasmodium falciparum* 3D7 strain was obtained from the insectary at the Walter Reed Army Institute of Research. Recombinant, full length *P. falciparum* CSP (3D7) was produced in *Escherichia coli* as previously described [15].

### Sporozoite dissection

Sporozoites (SPZ) were prepared by dissecting mosquitoes 16–20 days post blood feed using the Ozaki method [16], or the clean dissection method. The Ozaki method is a fast and simple dissection of mosquito heads followed by centrifugation of the material through a glass wool pillow; for the clean dissection method [17], salivary glands are removed from the mosquito heads, pooled and then disrupted by shear forces using a syringe and 18 g needle. SPZ were frozen as pellets and stored at − 80 °C to be used as lysates. Upon thawing, pellets were resuspended in PBS (pH 7.2), vigorously pipetted using siliconized/pre-lubricated pipette tips (to minimize loss of material), and lysis confirmed by microscopy.

### Memory B cell stimulation

Peripheral blood mononuclear cells (PBMC) from previously conducted clinical trials [IMRAS; Immunization by mosquito bite with radiation attenuated SPZ (NCT01994525) and MAL027 (NCT00075049)] were used. In the IMRAS trial, study participants received 5 doses of approximately 200 infectious bites/dose by *P. falciparum*-infected *Anopheles stephensi* mosquitoes. The goal of the trial was, apart from safety and immunogenicity, to develop reagents for the identification and validation of biomarkers of protection (Epstein et al. pers.commun.). In the MAL27 trial, study participants were immunized with three doses of RTS,S adjuvanted with AS01B or AS02A [18]. Cells from the MAL27 trial were used to represent ‘CSP-immune’ individuals. PBMC were thawed and counted using a Luna-FL™ Dual Fluorescence cell counter (fluorescence protocol with AO/PI to determine cell viability). Warm culture medium [(RPMI-1640 containing 10% fetal bovine serum (FBS), Pen/Strep, l-glutamine, non-essential amino acids (NEAA), Sodium Pyruvate, 2-mercaptoethanol] was prepared. Cell numbers were adjusted to 1.5 × 10^6^ cells/ml and stimulated with 1 μg/ml of the TLR7/8 ligand R848 (Mabtech) and 10 ng/ml recombinant human (rh) IL-2 (Mabtech) in culture medium for 36 to 48 h at 37 °C in a humidified incubator with 5% CO_2_ in six-well plates (Corning, Tewksbury, MA) Stimulating B cells polyclonally with R848 and supporting full activation with recombinant IL-2 ensures that all B cell clones are activated and produce immunoglobulins. Sporozoite material on the ELISpot plates enabled the capturing of malaria-specific antibodies secreted while incubating the cells in the ELISpot plates.

### Sporozoite-specific IgM/IgG ELISpot assay

PVDF-based membrane plates (MSIP, Millipore, Burlington, MA, USA) were activated with 15 µl/well 35% ethanol for 1 min following the manufacturer’s instructions. The plates were washed five times with tissue culture grade, sterile water. Next, the activated ELISpot plates were coated with 100 µl/well that equate to 30,000 lysed SPZ/well or recombinant CSP protein (1 µg/ml). The optimal amount of SPZ was determined in a previous study [11]. Assay controls were: (a) Bovine Serum Albumin (BSA, cell culture grade, Sigma Aldrich, St. Louis, MO, USA, 1 µg/ml) as the negative control to determine non-specific binding of antibodies to plates; (b) IgM-specific monoclonal antibody (mAb) MT11/12 (Mabtech) or IgG specific mAb MT91/145 (both at 15 µg/ml; Mabtech) as positive control since they capture any secreted antibody regardless of specificity. Plates were incubated overnight at 4 °C [11]. Liquid from wells coated with sporozoite lysate was gently removed and wells allowed to air dry since immobilizing the sporozoite material by air drying rather than fixation is superior regarding the reactivity with antibodies [11]. On the day of the experiment, plates were blocked with culture medium (RPMI 1640 with 10% FBS and supplements) for 2 h at 37 °C. It was necessary to use FBS in the culture medium since human AB pooled serum caused significant background issues. Next, R848-activated PBMC or CD19 enriched B cells were plated onto the ELISpot plates. The cell concentration of PBMC was 5 × 10^5^ cells/well (the maximum cell number recommended by the manufacturer of the ELISpot reagents) while the cell concentration of enriched B cells was 5 × 10^4^ to 10^5^. Cells were plated in triplicates. For B cell enrichment, R848-activated PBMC were used as starting material for the magnetic enrichment of B cells using CD19-microbeads (Miltenyi Biotec, Auburn, CA, USA) following the manufacturer’s instructions. In brief, polyclonally activated PBMC were harvested and cell numbers determined using a Luna-FL™ cell counter. Cells were pelleted (centrifugation at 300*g* for 10 min) and resuspended in degassed MACS buffer [PBS (pH 7.2) with 0.5% BSA and 2 mM EDTA] at a concentration of 10^7^ cells/100 µl. Twenty µl of anti-CD19 microbead-conjugated mAb was added and incubated for 15 min at 4 °C. Two ml of MACS buffer was then added and cells spun down to remove unbound antibody (300 g, 10 min). Finally, cells were resuspended in 500 µl MACS buffer and passed through MS columns (Miltenyi Biotec) inserted into an OctoMACS magnet (Miltenyi Biotec). Enriched cells were counted (Luna-FL™) and plated at a cell concentration of 5 × 10^4^ to 10^5^ cells/well. Plates were incubated for 24 h at 37 °C in a humidified incubator with 5% CO_2_ to allow for the capturing of sporozoite-specific antibodies. Cells were removed from the plates by washing (Biotek EL405 plate washer, Biotek Winooski, VT, USA) plates five times with 250 µl/well PBS. Detection of spots was achieved by adding biotinylated mAb MT22 (for IgM) or mAb MT78/145 (for IgG) at 1 µg/ml (100 µl /well; PBS with 0.5% FBS as diluent) for 2 h at room temperature. Plates were washed and incubated with alkaline phosphatase-conjugated Streptavidin–alkaline (1:1,000, PBS with 0.5% FBS as diluent). All reagents were obtained from Mabtech. After 1 h at room temperature, plates were washed and BCIP/NBT substrate added until spots were visible. Reaction was stopped by washing plates with tap water. Dried ELISpot plates were analysed using the AID Autoimmun Diagnostika GmbH ELISpot reader (Strassberg, Germany) and accompanying software.

### Statistical analysis

Statistically significant differences between the various assay conditions were determined by using two-sided T tests (Minitab 17, State College, PA, USA).

## Results and discussion

### CSP-specific B cells recognize sporozoites immobilized on ELISpot plates

**Fig. 1:**
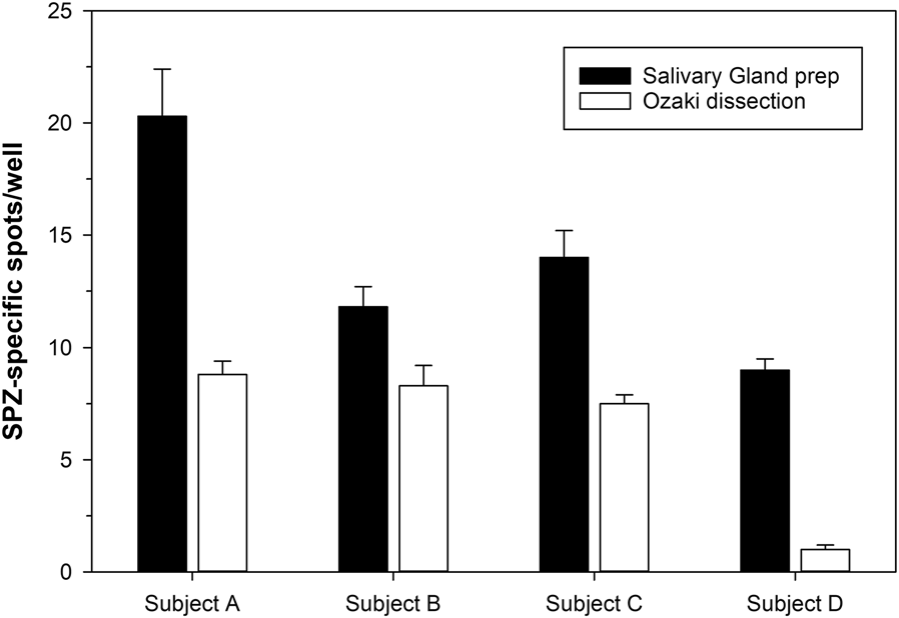
Impact of sporozoite preparation on signal strength. ELISpot plates were coated with lysates of sporozoite (30,000/well) either dissected using the Ozaki method (white bars) or by preparing salivary glands (black bars). 5 × 10^5^ PBMC/well from four different CSP-immune donors were plated. Data are expressed as the average number of sporozoite-reactive B cells (error bar represents standard deviation of triplicate wells)

A recent report described optimal assay conditions for a sporozoite-based ELISA [11]. In that study, monoclonal antibodies as well as sera from RAS-immune subjects recognized both, sporozoite lysates and intact SPZ. The results from that study demonstrated that, for convenience, sporozoite lysates can be used in lieu of freshly isolated SPZ. In the present study, ELISpot plates were coated under the same conditions with sporozoite lysate or with recombinant CSP (as positive control) or BSA (negative control). To determine the impact of the sporozoite preparation, SPZ were prepared by two different methods: (1) crude preparation using the Ozaki method [16]; (2) clean preparation by dissecting salivary glands from the mosquito heads. For the ease of performing the experiments and based on previous data obtained from the sporozoite-based ELISA, SPZ were isolated using both preparation techniques and then frozen for later use. PBMCs from four CSP-immune donors were plated at a cell concentration of 5 × 10^5^ cells/well and tested for reactivity (Fig. 1). The clean dissection provided stronger signals for all four donors. Therefore, all subsequent experiments were conducted using SPZ from cleanly dissected salivary glands. It is not clear why the crude sporozoite preparation yielded lower responses, but one possible explanation is that debris interferes with the reactivity of the antibodies.

### Optimization of assay sensitivity

**Fig. 2:**
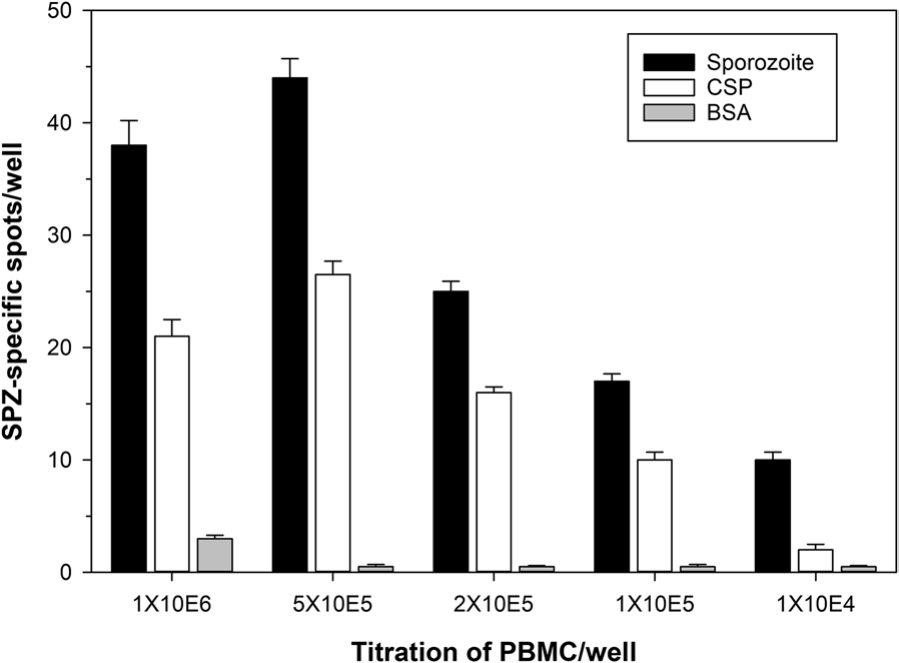
CSP-immune PBMC react with sporozoite lysates in an ELISpot format. Wells were either coated with sporozoite lysate (30,000/well, salivary gland preparation) or recombinant CSP protein (positive control) or BSA (negative control) and titrations of CSP-immune PBMC (indicated on x-axis) plated to determine the optimal cell concentration. Data (representative experiment) are expressed as the average number of reactive B cells (error represents SD of triplicate wells)

**Fig. 3:**
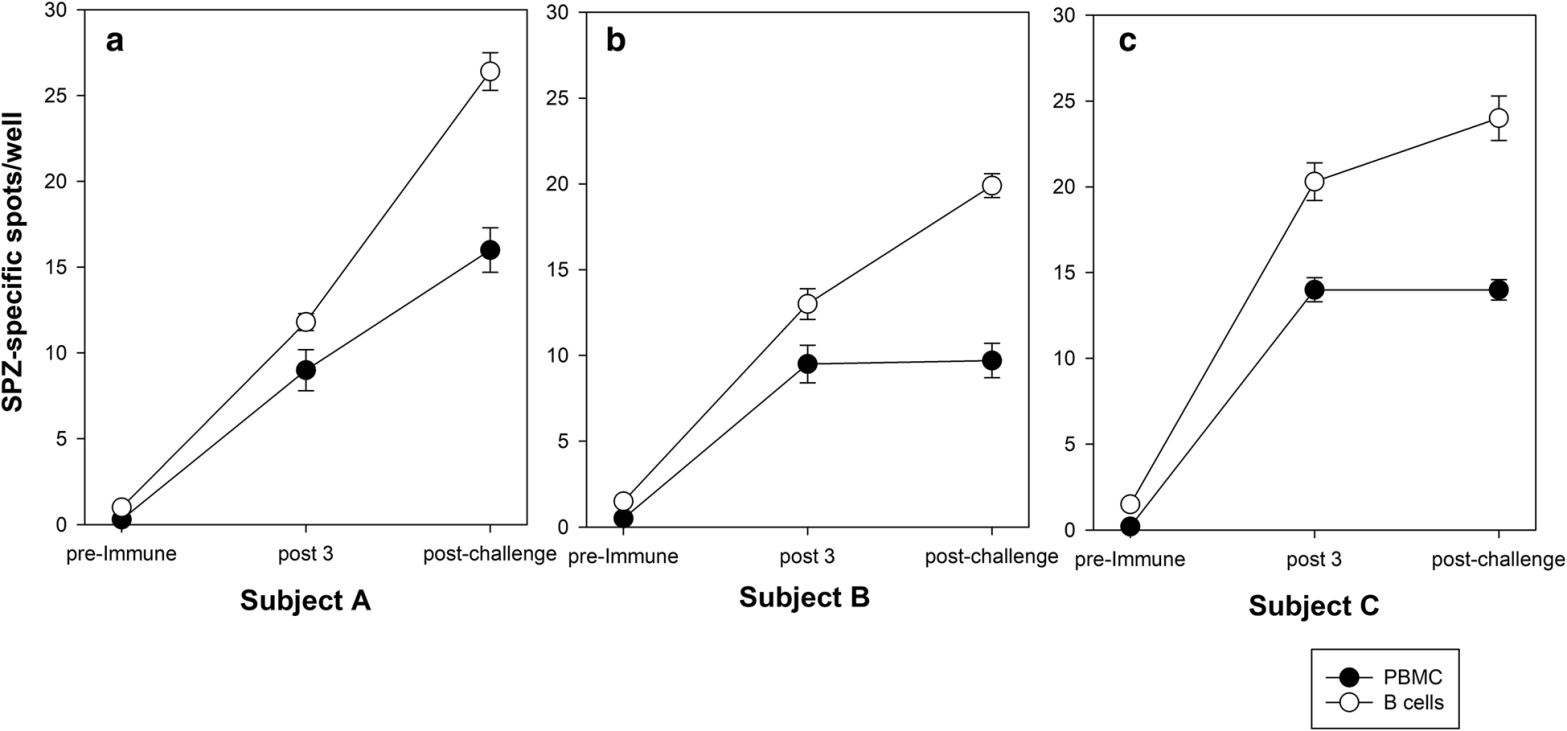
Enrichment of B cells prior to plating onto the sporozoite-ELISpot improves the assay sensitivity. Three CSP-immune donors (**a**, **b**, and **c** representing the donors) were tested to measure the parasite response for pre-immune, post 3 (i.e., pre challenge), and post challenge (4 weeks after controlled human malaria infection) time points. 5 × 10^5^ PBMC/well (black symbol) or 10^5^ purified B cells/well (white symbol) were plated. Data expressed as mean (SD) of triplicate wells

Initial experiments revealed that the signal strength was significantly dependent on the vaccine or malaria-exposure of the donors. To determine the detection threshold of the assay and to also increase the sensitivity of the assay, the following two experiments were conducted: (1) titre the cell number of CSP-immune PBMC to determine the dose response and detection threshold (Fig. 2); (2) compare the signal strength when using either CSP-immune PBMC or CSP-immune B cells in the assay (Fig. 3).

Titrating PBMC revealed that a linear response was achieved with cell numbers of between 10^5^ to 5 × 10^5^ cells/well. Adding more cells (10^6^ cells/well) did not increase signal strength. In contrast, plating a higher cell number than 5 × 10^5^ cells/well is not recommended by the manufacturer. A range of 2 × 10^5^ to 5 × 10^5^ is optimal and provides flexibility in cases where the sample volume is limited.

Human peripheral blood consists of 1% to 7% B cells. This raises the question whether purifying B cells with magnetically labelled CD19 could increase the sensitivity of the assay because it would be equivalent to plating a larger number of PBMCs (plating 10^5^ B cells would equate to 1.4–10 × 10^6^ PBMC), which is not possible due to crowding of cells in the well. Comparative experiments were set up whereby PBMCs versus positively enriched B cells (using CD19 microbeads) from each donor were tested for reactivity in the sporozoite-based ELISpot. Three volunteers from a previously conducted malaria clinical trial (RTS,S vaccine) were tested to assess parasite responses for pre-immune, post-3 (pre-challenge; 2 weeks after the last immunization) and post challenge (4 weeks after controlled human malaria infection) time points. Purified B cells showed higher spots per well as compared to unfractionated PBMCs (Fig. 3). The results demonstrated a statistically significant increase in the signal between purified B cells and PBMCs. Therefore, subsequent assays were always conducted with purified B cells after the polyclonal stimulation of PBMCs.

### Detection of parasite-specific, IgM and IgG-producing B cells

**Fig. 4:**
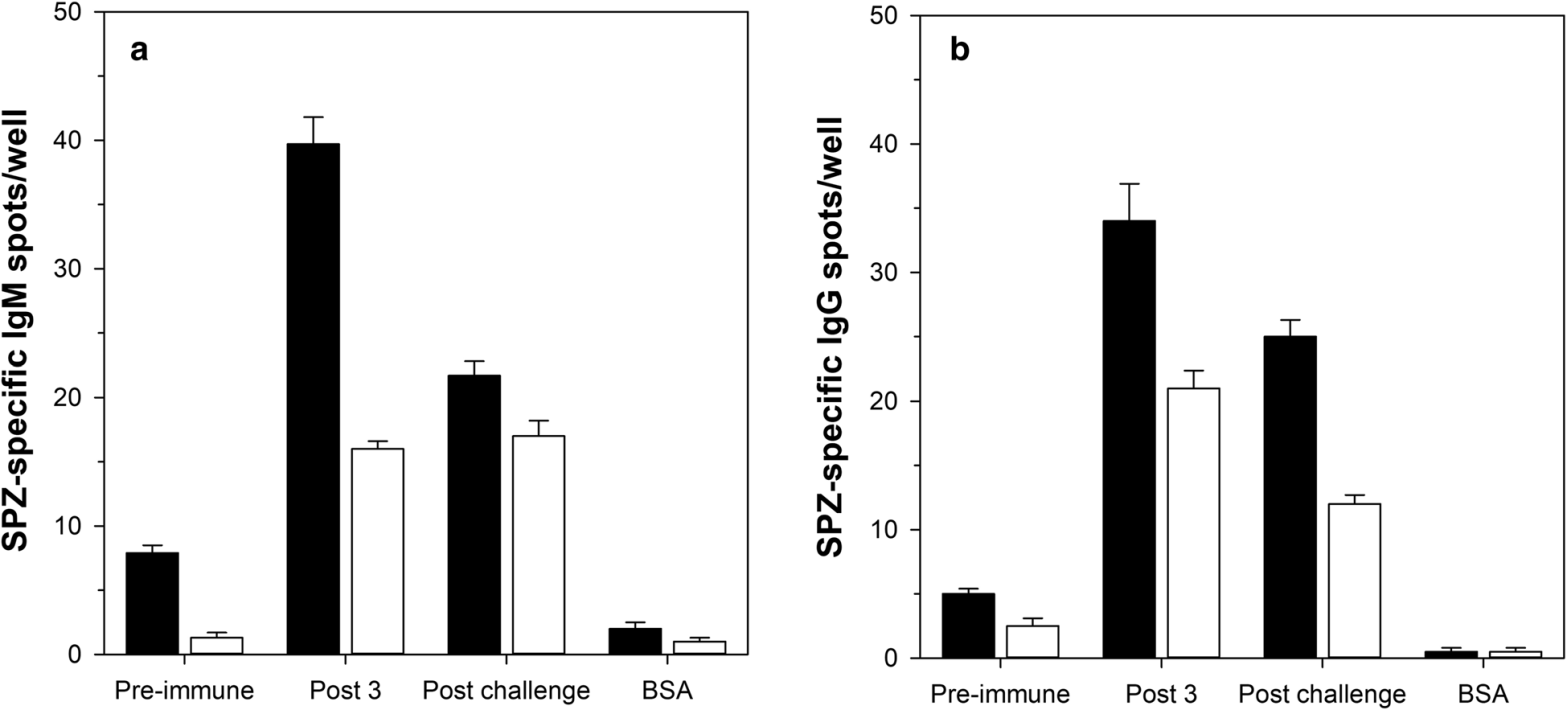
Detection of parasite-specific, IgM- and IgG-producing B cells. Parasite-specific IgM response (**a**). Parasite-specific IgG response (**b**). Data are represented as the mean number of sporozoite-specific spots per 10^5^ B cells (triplicate wells). Time points tested: pre-immune, post 3 (i.e., pre-challenge), post challenge (4 weeks after controlled malaria infection). BSA-coated (BSA) wells demonstrate non-specific binding of antibodies to the plate. Fill of bars indicates donors (n = 2)

The next step after establishing the primary conditions for the SPZ-based ELISpot assay was to determine the frequency of IgM and IgG producing B cells that recognize epitopes on *P. falciparum* SPZ. Pre-immune, post 3 (i.e., pre-challenge; 2 weeks post last immunization), and post challenge (4 weeks post controlled human malaria infection) time points from two different CSP-immune donors were tested for reactivity in the SPZ-based ELISpot. After the overnight incubation of purified B cells in the ELISpot plates, cells were removed and the plates either probed with biotinylated anti-human IgM or anti-human IgG monoclonal antibodies followed by Streptavidin-AP to assess the frequency of CSP-specific IgM or IgG producing B cells (Fig. 4). The results demonstrate that vaccination results in significant increases in the number of SPZ-specific IgM and IgG-producing B cells. A weak response was observed in pre-immune samples; this could reflect cross-reactivities of pre-existing antibodies to other pathogens such as *Toxoplasma*. Similarly, there is drop in the frequency of SPZ-specific B cells after a controlled human malaria infection (challenge). This could be due to the sequestration of SPZ-specific B cells in immunological organs and warrants further analysis.

### Application of sporozoite-specific B cell ELISpot protocol

**Fig. 5:**
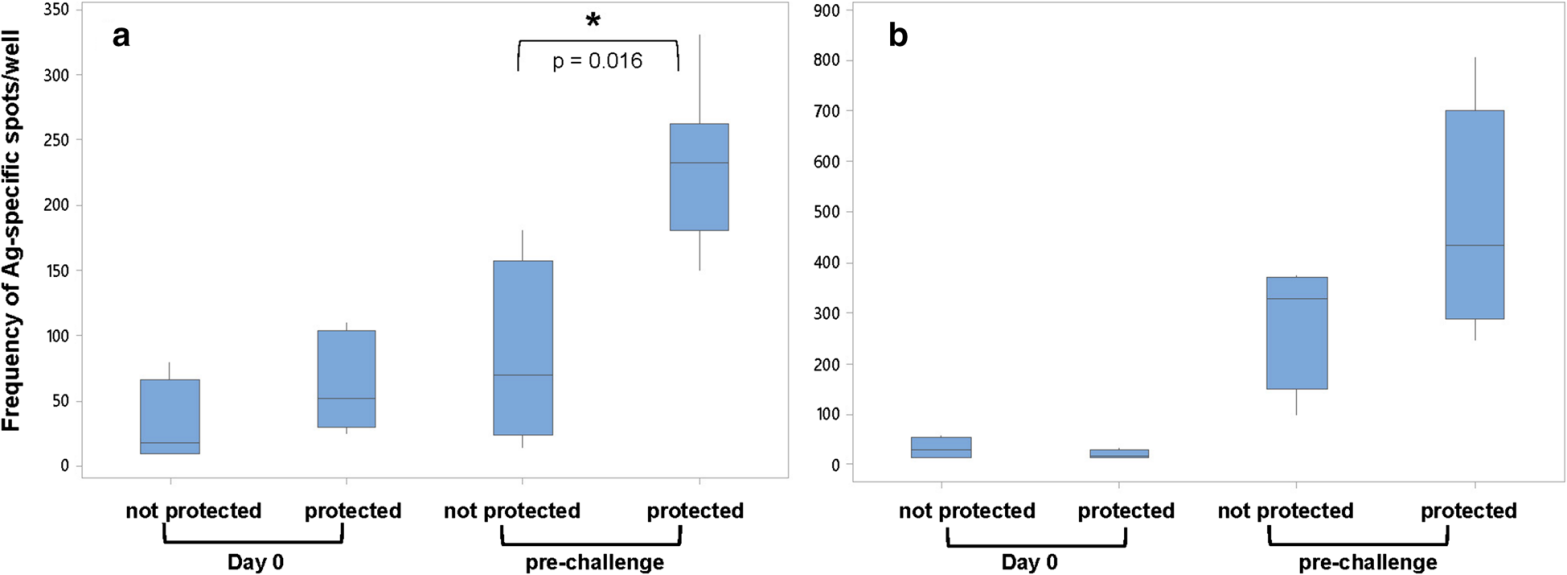
Sporozoite-ELISpot can discern between protected and non-protected RAS vaccinated subjects. Box plots represent the frequency of CSP-specific (**a**) or SPZ-specific (**b**) IgG-spots per 10^5^ plated B cells from protected (n = 8) and non-protected (n = 4) subjects per time point. Time points: pre-immune and pre-challenge (i.e., 2 weeks after last immunization, day of challenge)

The next step was to apply the SPZ-based ELISpot assay to human vaccine models by testing pre-immune and pre-challenge PBMCs from a RAS clinical trial under the established conditions (10^5^ purified B cells/well). Results were stratified based on the protective status of the donors (n = 8 for protected subjects and n = 4 for not-protected subjects); in the IMRAS trial, donors were immunized by receiving approx. 960 infectious bites of *P. falciparum* infected, irradiated *An. stephensi* mosquitoes and protective efficacy assessed in a controlled human malaria infection (CHMI). In the CHMI study, participants are challenged with five bites of *P. falciparum*-infected mosquitoes and evaluated daily for the presence of blood-stage parasites. Only donors that remain parasite-free after 28 days are considered protected (i.e., sterile protection). IgG responses were measured against SPZ and CSP (Fig. 5). The results provided several important observations: (a) the frequency of CSP-specific B cells is significantly lower compared to B cells reactive to SPZ. While the data confirm that CSP is one of the major antigens recognized by malaria-immune B cells, they also show that other sporozoite-specific antigens are present and recognized by a significantly higher number of B cells; (b) the frequency of CSP-specific B cells is significantly higher in subjects protected by the RAS vaccine (p = 0.016, 2-sided T-test); (c) the frequency of sporozoite-specific B cells has a strong trend to be higher in protected subjects (p = 0.08, 2-sided T-test). The latter observation suggests the utility of this readout for identifying immune correlates of protection. The role of CSP-specific antibodies, and therefore B cells, in mediating sterile protection has long been recognized [11, 15, 19, 20]. Not all antigens recognized by IMRAS-induced antibodies contribute to protection. The weaker correlation between frequency of sporozoite-specific B cells and protection (Fig. 5b) would suggest that some of the recognized antigens either do not correlate with protection or may even correlate with susceptibility.

## Conclusion

The successfully developed B-cell ELISpot assay measures the frequency of parasite-specific B cells. This assay is capable of detecting malaria-specific B cells secreting sporozoite-specific IgM and IgG in vitro. The newly developed assay shows high sensitivity and is robust enough to detect B cell responses induced by two distinct vaccine platforms in humans. This new immunoassay can assist in the evaluation of vaccine induced B-cell responses based on native sporozoite antigens and support the identification of immune correlates of protection against malaria infections.

## Data Availability

The data and detailed protocol can be made available upon request from the corresponding author.

## Abbreviations

BSA: bovine serum albumin
CSP: circumsporozoite protein
FBS: fetal bovine serum
GAS: genetically engineered sporozoites
HBsAg: hepatitis B surface antigen
IMRAS: immunization by mosquito bite with radiation attenuated sporozoites
mAb: monoclonal antibody
NEAA: non-essential amino acids
PBMC: peripheral blood mononuclear cells
RAS: radiation attenuated sporozoites
SPZ: sporozoite
WPV: whole parasite vaccines
